# Effects of ankle-foot orthosis stiffness on lower-limb joint work during various movement tasks in people with calf muscle weakness—an explorative study

**DOI:** 10.3389/fresc.2026.1994258

**Published:** 2026-10-27

**Authors:** Jack R. Roelfsema, Winfred Mugge, Niels F. J. Waterval

**Affiliations:** 1 Department of Biomechanical Engineering, Delft University of Technology, Delft, Netherlands; 2 Department of Rehabilitation Medicine, Amsterdam UMC Location University of Amsterdam, Amsterdam, Netherlands; 3 Amsterdam Movement Sciences, Rehabilitation and Development, Amsterdam, Netherlands

**Keywords:** ankle-foot orthosis, calf muscle weakness, gait initiation, impulse, joint work, obstacle crossing, propulsion, stiffness

## Abstract

**Background:**

Spring-like ankle–foot orthoses (AFOs) are provided to people with calf muscle weakness to improve mobility by supporting ankle push-off. During walking, a higher AFO stiffness reduces ankle work. However, other movement tasks pose other demands on the AFO. In this explorative study, we aimed to determine whether AFO presence and stiffness influence lower-limb joint-work differently during gait initiation, obstacle crossing, and step-up in individuals with calf muscle weakness.

**Methods:**

Seven participants with bilateral calf muscle weakness performed gait initiation, obstacle crossing, and step-up movements in a shoes-only condition and five AFO stiffness conditions (2.8–6.6 Nm/◦). Outcomes included forward and upward impulse, and (relative) positive lower-limb joint work. Trial-level linear mixed-effects models were used for analyses, with false discovery rate correction to account for multiple testing.

**Results:**

Compared with shoes-only, wearing an AFO (2.8 Nm/◦) did not affect forward or upward impulse. Absolute ankle and knee work reduced, while the relative contribution of knee work reduced and hip work increased when using an AFO. No interaction between using an AFO and movement task was found. Increasing AFO stiffness reduced absolute and relative positive ankle work (*q* *<* 0*.*001), while no effects on impulses nor on knee or hip work were found. Impulse and joint work significantly differed between movements, but no interaction stiffness-by-movement task interaction was found.

**Conclusion:**

Our explorative study indicates that an AFO of 2.8 Nm/◦ and increasing AFO stiffness further reduce absolute ankle work while no effect on impulse was found. For relative joint work our results indicate that an interaction might exist, where the effect of increasing AFO stiffness is more pronounced in activities with relatively higher ankle power demands.

## Introduction

Ankle–foot orthoses (AFOs) are commonly prescribed to improve mobility in individuals with calf muscle weakness (1–3). The effectiveness of AFOs is predominantly evaluated during level walking. Daily mobility, however, extends beyond walking and includes transitional and environmental movement tasks. Such movements impose other mechanical demands on the lower limbs and therefore the AFO (4, 5). Whether AFO effects observed during walking continue to apply during these daily movement tasks remains unclear.

Individuals with calf muscle weakness due to neuromuscular disorders have a reduced physical functioning, at least partly, due to a reduced ankle push-off work (6, 7). In healthy persons the timing and amount of ankle work is essential for performing safe and efficient movements, including walking (8–10). During walking ankle work generated during single stance elevates the center-of-mass upwards, thereby reducing the impact of contralateral heel-strike. Furthermore, single stance ankle work is important for stability by adjusting the center-of-mass velocity and precisely placing the foot (10). In case of calf muscle weakness, the reduced ankle push-off work must be compensated by increases in work at more proximal joints or the contralateral limb (11), which is mechanically less efficient. Furthermore, reduced ankle work negatively affects other aspects of daily life such as stability and accurate foot placement (12).

To support ankle push-off work and reduce the mechanical consequences of calf muscle weakness, spring-like AFOs are often provided, of which stiffness is a key determinant of effectiveness (13, 14). AFO stiffness can be defined as the resistance of the AFO to ankle rotation in the sagittal plane. With increasing AFO stiffness the maximal ankle dorsiflexion angle is reduced, which provides support and helps control tibia progression during walking (15). However, the resulting restriction in ankle range-of-motion negatively affects the muscle-tendon capacity to generate positive mechanical work during level walking (16, 17).

However, daily life consists of many more movement tasks for which ankle work is essential but pose other demands on the AFO. Gait initiation, obstacle crossing, and step-up are examples of such movement tasks which differ in the direction, timing, and joint-level distribution of mechanical output compared to level-ground walking. Gait initiation requires a larger forward impulse, usually generated by ankle push-off power, as the trailing limb accelerates the body forwards (5). Obstacle crossing increases ankle power requirements for both forward as well as upward acceleration to ascertain sufficient foot clearance and safe foot placement (4). Step-up movements require elevation of the body center of mass and, besides placing a higher demand on the ankle joint also substantially increases knee and hip extensor demands to generate the upward impulse.

It is unknown to what extent the findings that AFOs and a higher AFO stiffness limit push-off and ankle work are generalizable to other movement tasks. Exploration whether there is an interaction effect between AFO and movement task is warranted to determine the usability of AFO. Therefore the aim of this explorative study was to determine whether the effects of AFOs and AFO stiffness on impulse, absolute joint work and joint work distribution interact with movement task, including gait initiation, obstacle crossing, and step-up in individuals with calf muscle weakness. We hypothesized that forward and upward impulse do not differ between AFO stiffness conditions, reflecting preservation of task-level mechanical output. We further hypothesize that increasing stiffness results in larger reductions in absolute and relative ankle work in tasks in which trailing-limb ankle push-off contributes more strongly to propulsion, particularly gait initiation and obstacle crossing.

## Materials and methods

### Participants

This study was an addendum to the ADJUST-AFO study, of which the protocol was approved by the Medical Ethics Committee of Amsterdam UMC (Amsterdam, The Netherlands) and registered in the Dutch Trial Register (NL-OMON53208). All participants provided written informed consent prior to participation.

Inclusion criteria of the ADJUST-AFO study were:

- Age >18 years old
- Body mass <120 kg;
- Presence of uni- or bilateral calf muscle weakness
- Able to walk for at least 20 min with an AFO, with walking aids if necessary

Exclusion criteria were:

- Use of a Cardiac Implantable Electronic Device (CIED);
- Exhibited pes equinus under weight-bearing conditions;

For the additional measurement we only included participants with bilateral weakness who were able to walk safely and independently with the experimental AFO. Unilateral AFO users have a strong preference to push-off with their non-affected leg, when performing activities like step-up. Hence, studying how AFOs affect their push-off during such activities is less relevant.

Based on these criteria we were able to include seven participants, consisting of 4 males and 3 females. Participants were on average 65.7 (range: 34–76) years old, had a height of 177 (SD: 8) cm, body mass of 74.5 (SD:1 0.1) kg and BMI of 23.6 (SD: 2.9) kg/m^2^. Participants were diagnosed with Charcot-Marie-Tooth disease (CMT, *n* = 3), Chronic Idiopathic Axonal Polyneuropathy (CIAP, *n* = 2), post-polio syndrome and unspecified lower leg weakness. All but one were experienced dorsal leaf AFO users.

### Ankle-foot orthosis and stiffness conditions

Measurements were performed in a shoes-only condition as well as with 5 AFO stiffness conditions (stiffness range: 2.8, 3.5, 4.3, 5.3, and 6.6 Nm/◦). For the shoes-only condition participants used standardized footwear. The same footwear was used in combination with a modular spring-like ankle–foot orthosis (AFO) for the AFO conditions (Figure 1). The modular AFO consisted of a carbon-fiber footplate, calf shell, and interchangeable posterior leaf spring element, as used in previous studies (Figure 1) (15). AFO stiffness was modulated by exchanging the posterior leaf spring.

**Figure 1:**
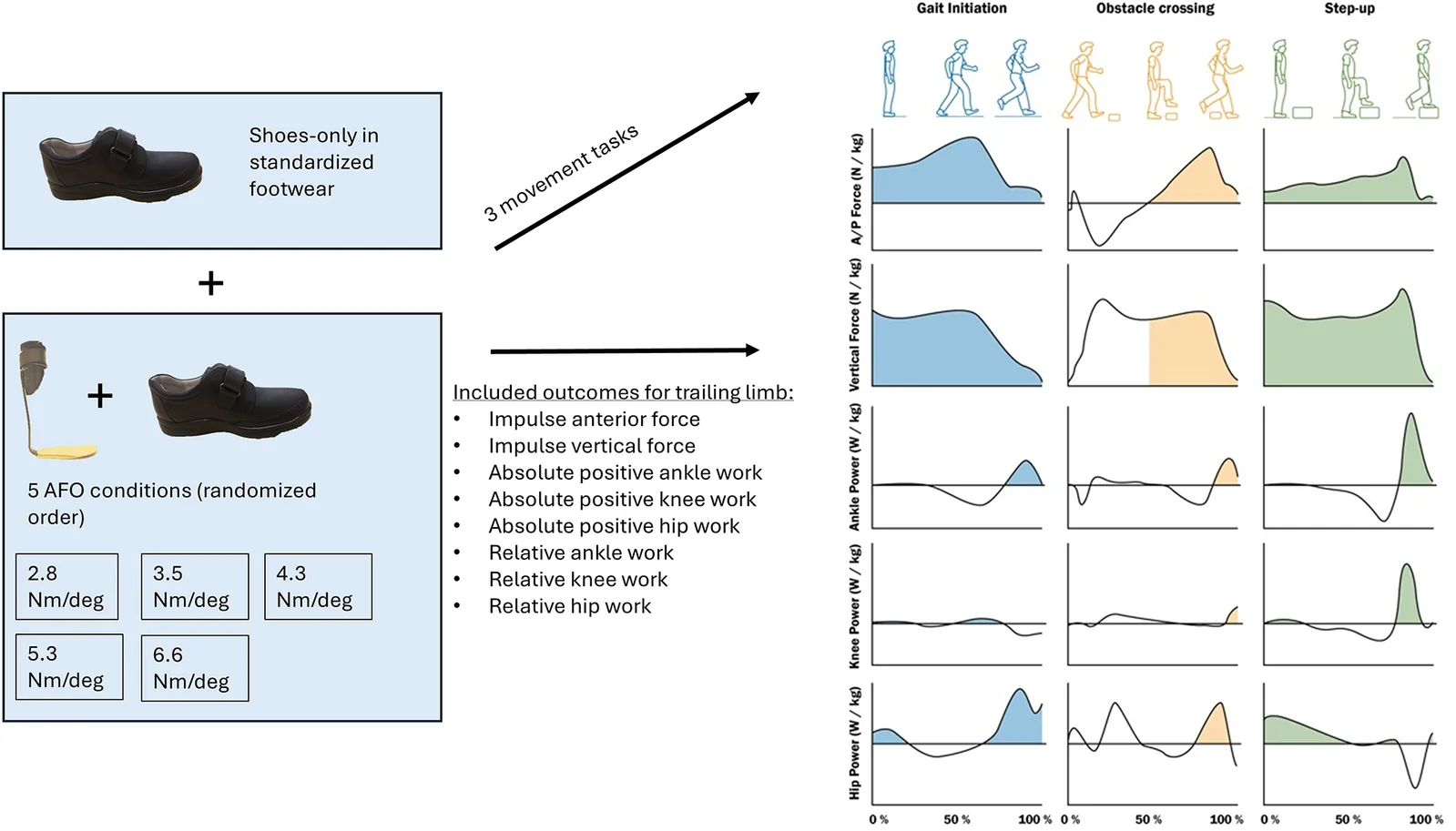
Diagram summarizing the experiment, providing an overview of the conditions, movement tasks and outcome measures.

### Experimental procedures

Measurements were first performed in the shoes-only condition. Subsequently, the five AFO stiffness conditions were tested in randomized order. For each condition, per protocol participants performed the following tasks: gait initiation, obstacle crossing, step-up, sit-to-stand, step-down, standing, and a Timed Up and Go test. The present study only includes data on gait initiation, obstacle crossing, and step-up because these movement tasks contain identifiable propulsion phases and well-defined single-limb force-plate contact suitable for joint-level mechanical work analysis.

During the movement tasks three-dimensional kinematic data were collected using a motion capture system (version 2.12 Vicon Nexus, Vicon, Oxford Metrics, UK). Reflective markers were placed according to the Plug-in Gait marker model. Marker trajectories were sampled at 100 Hz and filtered in Vicon Nexus using a Woltring smoothing spline with a mean squared error (MSE) value of 10. Ground reaction forces were recorded using force plates embedded in the laboratory floor (AMTI OR6-7, Watertown, MA USA; 464 × 508 × 82.5 mm) at a sampling frequency of 1000 Hz.

Participants received standardized movement instructions. Before the shoes-only condition one practice trial for each movement task was performed. Additional practice trials were allowed when requested by the participant or deemed necessary by the investigator. Each movement task was repeated until at least three successful trials per condition per task were obtained. Where this was not achievable, the maximum available trials were retained. Participants were allowed rest between trials and conditions as needed. No familiarization with the different AFO conditions was given due to the substantial time burden of the whole protocol.

Gait initiation was performed from standing with the feet comfortably next to each other in the frontal plane. Participants stood with one foot on each of two adjacent force plates. Upon a verbal countdown (“3–2–1–go”) given by the investigator, participants initiated walking at a self-selected pace and continued walking for three meters. A third force plate was positioned anterior to and centered between the two initial force plates to identify the first step after gait initiation. To be considered successful, the foot of the leading limb had to have complete and isolated force plate contact. Participants were not restrained regarding their step length or speed.

Obstacle crossing was performed during walking along a 12-meter level walkway instrumented with force plates. A rigid beam-shaped obstacle (100 × 10 × 18.5 cm; width  ×  height  ×  depth) was positioned between two force plates. Upon a verbal countdown (“3–2–1–go”) given by the investigator, participants were instructed to walk at a comfortable speed along the walkway and step over the obstacle as naturally as possible. A trial was considered successful when complete and isolated force-plate contact was obtained for the trailing limb prior to obstacle clearance.

Step-up was performed, similar as gait initiation, from standing with the feet comfortably next to each other in the frontal plane. Participants stood with one foot on each of two adjacent force plates. Upon a verbal countdown (“3–2–1–go”) given by the investigator, participants ascended a platform (90 ×  36 ×  15 cm; width  ×  height  ×  depth) using a step-over-step strategy, first placing the leading limb on the platform and subsequently bringing the trailing limb onto the platform. No force plates were placed on the platform. A trial was considered successful when isolated force plate contact was obtained for the trailing limb during push-off and the participant achieved stable bilateral stance on the platform. Hand support by a lab technician to maintain balance was allowed.

### Outcome measures and data processing

As outcome measures, we quantified propulsive impulse and both absolute and relative positive joint work of the trailing limb during the propulsive phase. The forward impulse and upward impulse provide measures of push-off force. Joint work provides a joint-specific measure of mechanical energy. Relative joint work characterizes the distribution of positive mechanical work across the ankle, knee, and hip, while reducing the influence of between-trial differences in total lower-limb work (Figure 1).

For all three movement tasks, joint kinematics and kinetics were quantified in Vicon Nexus using the Dynamic Plug-in Gait pipeline. The impulses and work were normalized to body mass. Forward and upward impulses (in *N* · *s/kg)* were determined through numerical integration using the trapezoidal rule of the corresponding ground reaction force component for the corresponding propulsive phase. Normalized positive work in *J/kg* at each joint was calculated by integrating the positive portion of the corresponding joint power during the same propulsion phase. Relative joint work was expressed as percentage of the total work produced by ankle, knee and hip together.

The propulsion phase was defined as follows for the three movement tasks:

*Gait initiation:* defined as the interval from lead-limb toe-off to trailing-limb toe-off. This interval captures the period in which the trailing limb provides the primary mechanical contribution during the transition from standing to forward progression (13).
*Obstacle crossing:* defined from the mid-stance minimum in vertical ground reaction force until toe-off of the trailing limb. This window was selected to capture the push-off phase while excluding the preceding loading and braking phases, consistent with the force profile characteristics of trailing-limb stance during obstacle crossing (18, 19).
*Step-up:* defined as the interval from lead-limb toe-off to trailing-limb toe-off. This interval captures the trailing-limb contribution to upward progression toward the platform (21).

From these movements we also determined step length, step width and toe-clearance. Step length was calculated as the anterior–posterior distance between the trailing-limb toe marker and the leading-limb heel marker at the moment of leading-limb heel-contact. At the same timepoint the step width was calculated as the medio-lateral distance between the heel-markers. For obstacle crossing and step-up lead- and trail-limb toe clearance was calculated. Clearance was defined as the minimum vertical distance between the toe marker and the upper surface of the obstacle or step.

### Statistical analyses

We performed two main analyses; the effect of the most flexible AFO (2.8 Nm/◦)and the effect of further increasing AFO stiffness on impulse and joint work. For both analyses we used linear mixed-effects models (LMMs) in order to test across multiple stiffness levels and movement tasks in one test. Analyses were conducted at the trial level to retain within-subject variability and account for repeated measurements within participants. For both the analysis of an AFO (2.8 Nm/◦) and AFO-stiffness analyses, we followed a standardized random-effects model selection procedure**.** Models were rejected if they failed to fit, fitting generated invalid or extreme random-effect variance estimates (<0 or >10,000), or coefficient estimates were highly unstable (|SE/estimate| > 100). We initially evaluated a correlated participant-specific random-effects structure, followed by an uncorrelated version of the same movement-specific random-effects structure and a simpler model containing a general participant-specific random slope for stiffness. As they all failed to convergence for all outcomes measures the final random-intercept model [(1 | Subject)] was retained.

All LMMs were fitted using the lme4 package (25), and fixed-effect hypothesis tests were obtained using the lmerTest package with Satterthwaite approximation for denominator degrees of freedom (26). Statistical analyses were performed in R (R Foundation for Statistical Computing, Vienna, Austria).

In this analysis the trials are nested within the subject and not treated as independent variables. The LMMs were fitted using the lme4 package (25), and fixed-effect tests were obtained using the lmerTest package with Satterthwaite approximation for denominator degrees of freedom (26). All statistical analyses were performed in R (R Foundation for Statistical Computing, Vienna, Austria).

**Effect of AFO of 2.8 Nm/**◦**:** To evaluate the effect of wearing an AFO, the shoes-only condition was compared with the lowest AFO stiffness condition (2.8 Nm/◦). For each primary outcome, an LMM was fitted with presence of the AFO of 2.8 Nm/◦ (binary outcome), movement task (categorical outcome; gait initiation, obstacle crossing, and step-up), and their interaction as fixed effects. The AFO 2.8 Nm/◦ ×  movement task interaction was used to assess whether the effect of an AFO differed between movement tasks.
**AFO stiffness analysis:** To evaluate effects of AFO stiffness, the five AFO stiffness conditions were analyzed separately from the shoes-only condition. For each outcome, an LMM was fitted with stiffness, movement task, and their interaction as fixed effects. Stiffness was treated as a continuous variable in Nm/◦, allowing estimation of a linear response across the tested stiffness range. A linear response was expected as previous simulations and experimental work showed such a trend in the stiffness range measured during that study (18).

For the analysis with the AFO of 2.8 Nm/◦ 141 trials were available. For the stiffness analysis, 361 trials from the five AFO stiffness conditions were included. Across all included trials, the distribution over movement tasks was 144 for gait initiation, 148 for obstacle crossing, and 140 for step-up. For the shoes-only condition, four trials of obstacle crossing were excluded from analysis as no isolated single-limb force-plate contact was obtained.

To control for multiple testing across outcomes, false discovery rate (FDR) correction was applied using the Benjamini–Hochberg procedure (27) separately for the effect of an AFO of 2.8 Nm/◦ and AFO stiffness analyses. Corrections were applied within two families per analysis: the main model tests (main effects and interaction), across all eight primary outcomes, and the movement-specific *post-hoc* effects (Tables 1, 2), across all outcome–task combinations (24 tests). Adjusted *p*-values are reported as q-values, with q < 0.05 considered significant.

**Table 1:** Movement-specific effects of AFO with stiffness of 2.8 Nm/degree on biomechanical outcomes. Values are reported as median [IQR] for impulse outcomes and mean (SD) for joint work outcomes.

Outcome	Shoes-only	2.8 Nm/degree	Beta (e.g., effect per 1 Nm/deg increase in stiffness)
Gait initiation			
Forward impulse (N·s/kg)	0.68 [0.61−0.74]	0.70 [0.66–0.73]	−0.00406 [−0.0635 to 0.0554]
Upward impulse (N·s/kg)	5.19 [4.89–5.94]	5.40 [4.83–5.52]	−0.29 [−0.98 to 0.41]
Ankle relative positive work (%)	26.7 (8.9)	30.4 (9.2)	3.33 [−1.01 to 7.67]
Knee relative positive work (%)	28.3 (14.0)	12.0 (7.0)	−12.81 [−20.40 to −5.21]
Hip relative positive work (%)	45.0 (16.2)	57.6 (9.0)	10.01 [2.55 to 17.46]
Ankle positive work (J/kg)	0.090 (0.051)	0.079 (0.033)	−0.0136 [−0.0376 to 0.0103]
Knee positive work (J/kg)	0.094 (0.051)	0.030 (0.016)	−0.0516 [−0.0992 to −0.00392]
Hip positive work (J/kg)	0.146 (0.061)	0.154 (0.061)	−0.00129 [−0.0292 to 0.0267]
Obstacle crossing			
Forward impulse (N·s/kg)	0.47 [0.40–0.52]	0.41 [0.38–0.49]	−0.0330 [−0.0936 to 0.0276]
Upward impulse (N·s/kg)	3.42 [3.23–3.93]	3.92 [3.73–4.32]	−0.17 [−0.88 to 0.54]
Ankle relative positive work (%)	37.5 (12.0)	40.3 (5.7)	2.13 [−2.06 to 6.33]
Knee relative positive work (%)	43.6 (18.7)	32.8 (14.5)	−10.87 [−18.21 to −3.53]
Hip relative positive work (%)	18.8 (8.4)	27.0 (11.7)	8.59 [1.50 to 15.69]
Ankle positive work (J/kg)	0.146 (0.061)	0.109 (0.023)	−0.0366 [−0.0597 to −0.0136]
Knee positive work (J/kg)	0.183 (0.119)	0.091 (0.050)	−0.0946 [−0.14 to −0.0486]
Hip positive work (J/kg)	0.072 (0.042)	0.074 (0.044)	0.00582 [−0.0214 to 0.0331]
Step-up			
Forward impulse (N·s/kg)	0.50 [0.46–0.57]	0.52 [0.45–0.56]	0.0560 [−0.00345 to 0.12]
Upward impulse (N·s/kg)	8.20 [7.95–10.64]	8.20 [7.30–8.68]	−0.43 [−1.12 to 0.27]
Ankle relative positive work (%)	29.9 (12.6)	28.9 (5.7)	−1.89 [−6.24 to 2.46]
Knee relative positive work (%)	42.4 (15.6)	42.8 (12.7)	−0.42 [−8.02 to 7.18]
Hip relative positive work (%)	27.7 (9.6)	28.3 (14.8)	1.73 [−5.76 to 9.22]
Ankle positive work (J/kg)	0.136 (0.059)	0.130 (0.050)	−0.0103 [−0.0343 to 0.0138]
Knee positive work (J/kg)	0.218 (0.109)	0.206 (0.099)	−0.0203 [−0.0679 to 0.0274]
Hip positive work (J/kg)	0.123 (0.039)	0.109 (0.034)	−0.00964 [−0.0376 to 0.0183]

**Table 2:** Movement-specific effects of AFO stiffness on biomechanical outcomes. Values are reported as median [IQR] for impulse outcomes and mean (SD) for joint work outcomes. The beta reflects of the effect of a 1 Nm/degree increase in stiffness, including its confidence interval.

Outcome	2.8 Nm/degree	3.5 Nm/degree	4.3 Nm/degree	5.3 Nm/degree	6.6 Nm/degree	Beta (e.g., effect per 1 Nm/deg increase in stiffness)
Gait initiation						
Forward impulse (N·s/kg)	0.70 [0.66–0.73]	0.71 [0.65–0.73]	0.71 [0.68–0.73]	0.69 [0.67–0.70]	0.70 [0.67–0.74]	0.00225 [−0.00645 to 0.0110]
Upward impulse (N·s/kg)	5.40 [4.83–5.52]	5.23 [4.95–5.32]	5.14 [4.67–5.51]	5.27 [4.75–5.75]	5.27 [4.60–5.69]	0.0541 [−0.0852 to 0.19]
Ankle relative positive work (%)	30.4 (9.2)	29.0 (9.3)	28.5 (9.6)	25.2 (7.4)	24.5 (7.7)	−1.69 [−2.39 to −1.00]
Knee relative positive work (%)	12.0 (7.0)	12.3 (8.7)	11.6 (8.4)	13.3 (6.4)	13.6 (5.8)	0.38 [−1.10 to 1.86]
Hip relative positive work (%)	57.6 (9.0)	58.7 (9.4)	59.9 (10.6)	61.5 (7.6)	61.9 (7.7)	1.31 [−0.33 to 2.95]
Ankle positive work (J/kg)	0.079 (0.033)	0.076 (0.027)	0.077 (0.027)	0.062 (0.019)	0.064 (0.035)	−0.00508 [−0.00828 to −0.00187]
Knee positive work (J/kg)	0.030 (0.016)	0.031 (0.020)	0.031 (0.021)	0.032 (0.015)	0.034 (0.014)	0.00026 [−0.00718 to 0.00770]
Hip positive work (J/kg)	0.154 (0.061)	0.155 (0.042)	0.167 (0.054)	0.153 (0.041)	0.159 (0.044)	0.00006 [−0.00477 to 0.00488]
Obstacle crossing						
Forward impulse (N·s/kg)	0.41 [0.38–0.49]	0.40 [0.31–0.44]	0.40 [0.31–0.41]	0.39 [0.30–0.41]	0.40 [0.35–0.43]	−0.00795 [−0.0173 to 0.00143]
Upward impulse (N·s/kg)	3.92 [3.73–4.32]	3.61 [3.02–4.00]	3.38 [3.15–3.84]	3.73 [3.48–4.44]	3.90 [3.67–3.98]	−0.00445 [−0.15 to 0.15]
Ankle relative positive work (%)	40.3 (5.7)	40.0 (8.4)	38.2 (5.4)	35.3 (7.9)	32.4 (7.7)	−1.96 [−2.71 to −1.22]
Knee relative positive work (%)	32.8 (14.5)	32.2 (14.1)	32.9 (13.6)	37.9 (12.3)	37.0 (15.5)	1.22 [−0.37 to 2.81]
Hip relative positive work (%)	27.0 (11.7)	27.8 (11.3)	28.9 (14.9)	26.8 (10.8)	30.6 (10.5)	0.75 [−1.00 to 2.50]
Ankle positive work (J/kg)	0.109 (0.023)	0.103 (0.023)	0.097 (0.020)	0.085 (0.023)	0.081 (0.028)	−0.00811 [−0.0116 to −0.00465]
Knee positive work (J/kg)	0.091 (0.050)	0.084 (0.040)	0.084 (0.041)	0.094 (0.036)	0.094 (0.040)	−0.00013 [−0.00812 to 0.00786]
Hip positive work (J/kg)	0.074 (0.044)	0.075 (0.043)	0.074 (0.046)	0.068 (0.041)	0.078 (0.042)	0.00029 [−0.00490 to 0.00549]
Step-up						
Forward impulse (N·s/kg)	0.52 [0.45–0.56]	0.52 [0.41–0.53]	0.50 [0.46–0.51]	0.51 [0.42–0.55]	0.48 [0.41–0.55]	0.00286 [−0.00615 to 0.0119]
Upward impulse (N·s/kg)	8.20 [7.30–8.68]	7.68 [7.32–8.11]	7.92 [7.42–8.50]	7.72 [7.25–8.87]	7.99 [7.02–8.31]	0.19 [0.0443 to 0.33]
Ankle relative positive work (%)	28.9 (5.7)	26.9 (6.8)	26.7 (6.0)	28.3 (5.7)	25.1 (5.8)	−0.47 [−1.19 to 0.25]
Knee relative positive work (%)	42.8 (12.7)	42.5 (14.8)	38.4 (11.2)	38.3 (11.1)	44.0 (11.8)	−0.15 [−1.68 to 1.38]
Hip relative positive work (%)	28.3 (14.8)	30.5 (15.9)	34.9 (15.4)	33.3 (13.7)	30.9 (11.6)	0.65 [−1.04 to 2.34]
Ankle positive work (J/kg)	0.130 (0.050)	0.119 (0.053)	0.110 (0.056)	0.123 (0.050)	0.094 (0.040)	−0.00593 [−0.00925 to −0.00261]
Knee positive work (J/kg)	0.206 (0.099)	0.196 (0.104)	0.160 (0.080)	0.178 (0.098)	0.174 (0.093)	−0.00716 [−0.0149 to 0.00052]
Hip positive work (J/kg)	0.109 (0.034)	0.115 (0.042)	0.121 (0.043)	0.128 (0.036)	0.104 (0.030)	0.00028 [−0.00471 to 0.00528]

For each model, fixed-effect estimates, F-statistics, *p*-values, and FDR-adjusted q-values were extracted. Movement-specific stiffness slopes were extracted to describe movement task-specific response patterns. Normally distributed data are presented as mean (SD), while non-normally distributed data are presented as median [InterQuartile Range].

## Results

### Analysis shoe versus AFO of 2.8 nm/◦

The AFO of 2.8 Nm/◦ did not significantly affect forward (*F*(1*,* 122*.*6) = 0*.*13, *p* = 0*.*717, *q* = 0.820) or upward impulse (*F*(1*,* 123*.*5) = 2*.*04, *p* = 0*.*156, *q* = *0.249*). Both impulse outcomes differed significantly between movement task (both *q* *<* 0*.*001). No statistically significant AFO 2.8 Nm/◦ × movement task interactions were found (Figures 2, 3).

**Figure 2:**
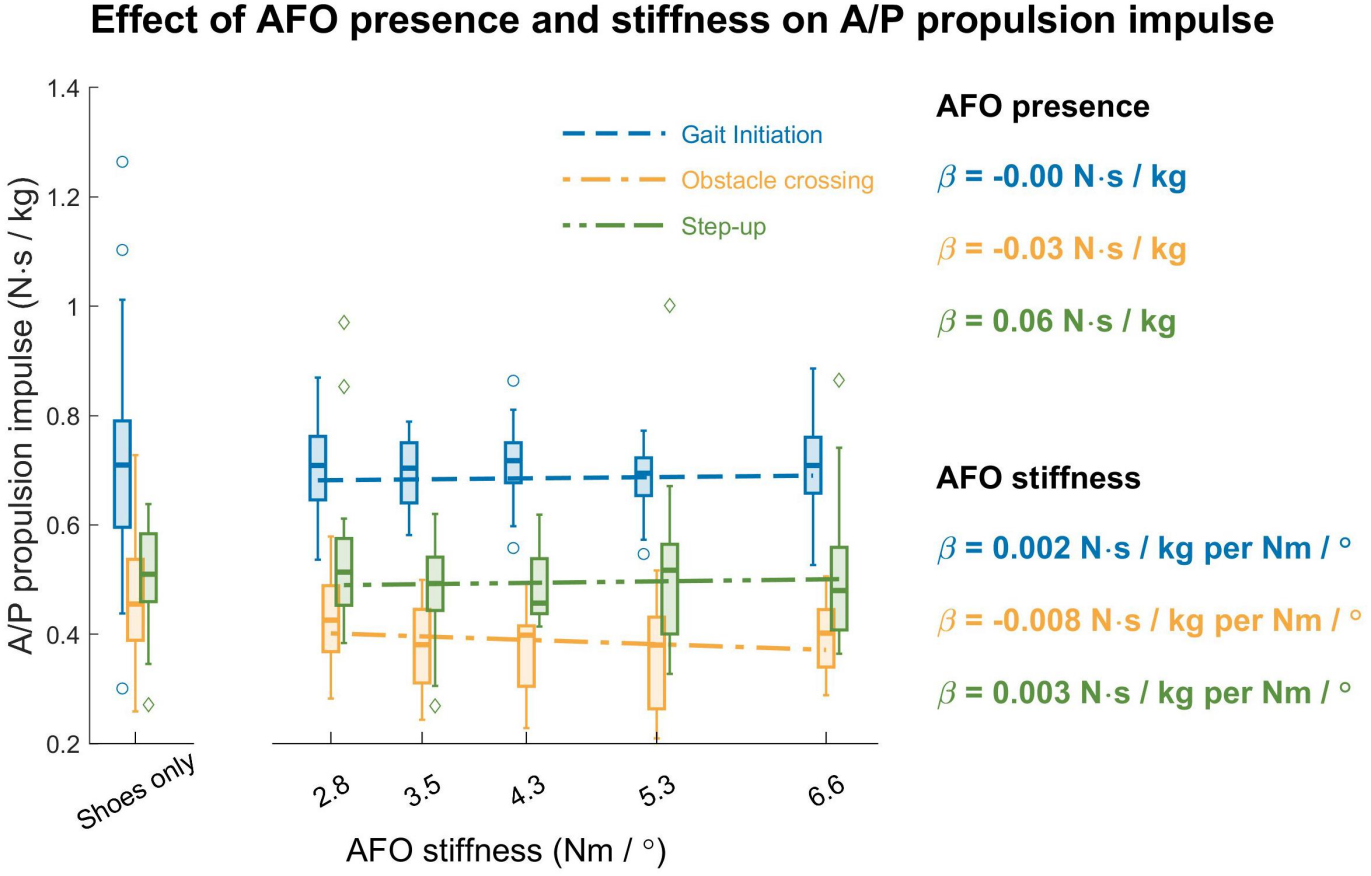
Effect of AFO and AFO stiffness on anterior-posterior propulsive impulse.

**Figure 3:**
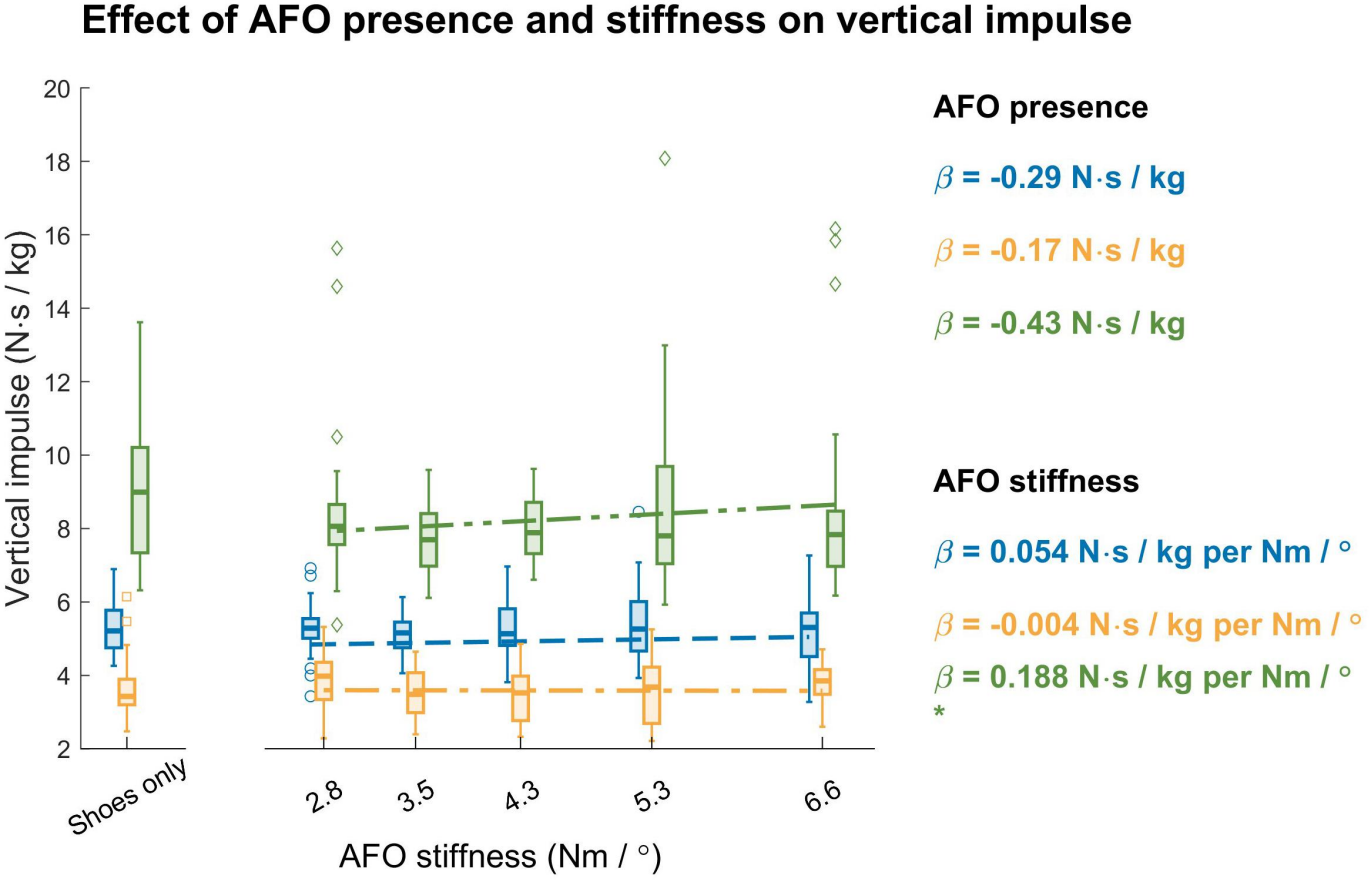
Effect of AFO and AFO stiffness on vertical impulse.

Regarding absolute joint work, an AFO of 2.8 Nm/◦ reduced positive ankle work (F(1,129.1) = 8.49, *p* = 0.0042, *q* = 0.0084) and absolute positive knee work (F(1,125.4) = 16.23, *p* *<* 0.0001, *q* *=* *0.0008*) (Figures 4, 5). No effect of this AFO on positive hip work was detected (F(1,6) = 0.037, *p* = 0.8546, *q* = 0.8546) (Figure 6). For all three joints a significant effect of movement task on absolute positive work was found (q < 0.001 for all). Ankle and knee work were highest during step-up, while hip work was highest during gait initiation. No significant interaction between the AFO of 2.8 Nm/◦ and movement task was found for the ankle, knee or hip joint work (q > 0.2930).

**Figure 4:**
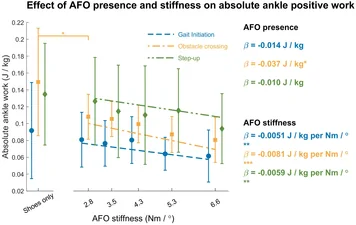
Effect of AFO and AFO stiffness on absolute ankle positive work.

**Figure 5:**
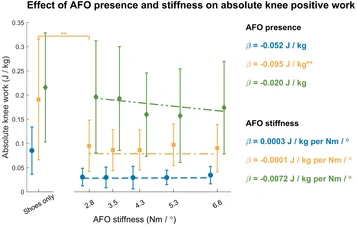
Effect of AFO and AFO stiffness on absolute knee positive work.

**Figure 6:**
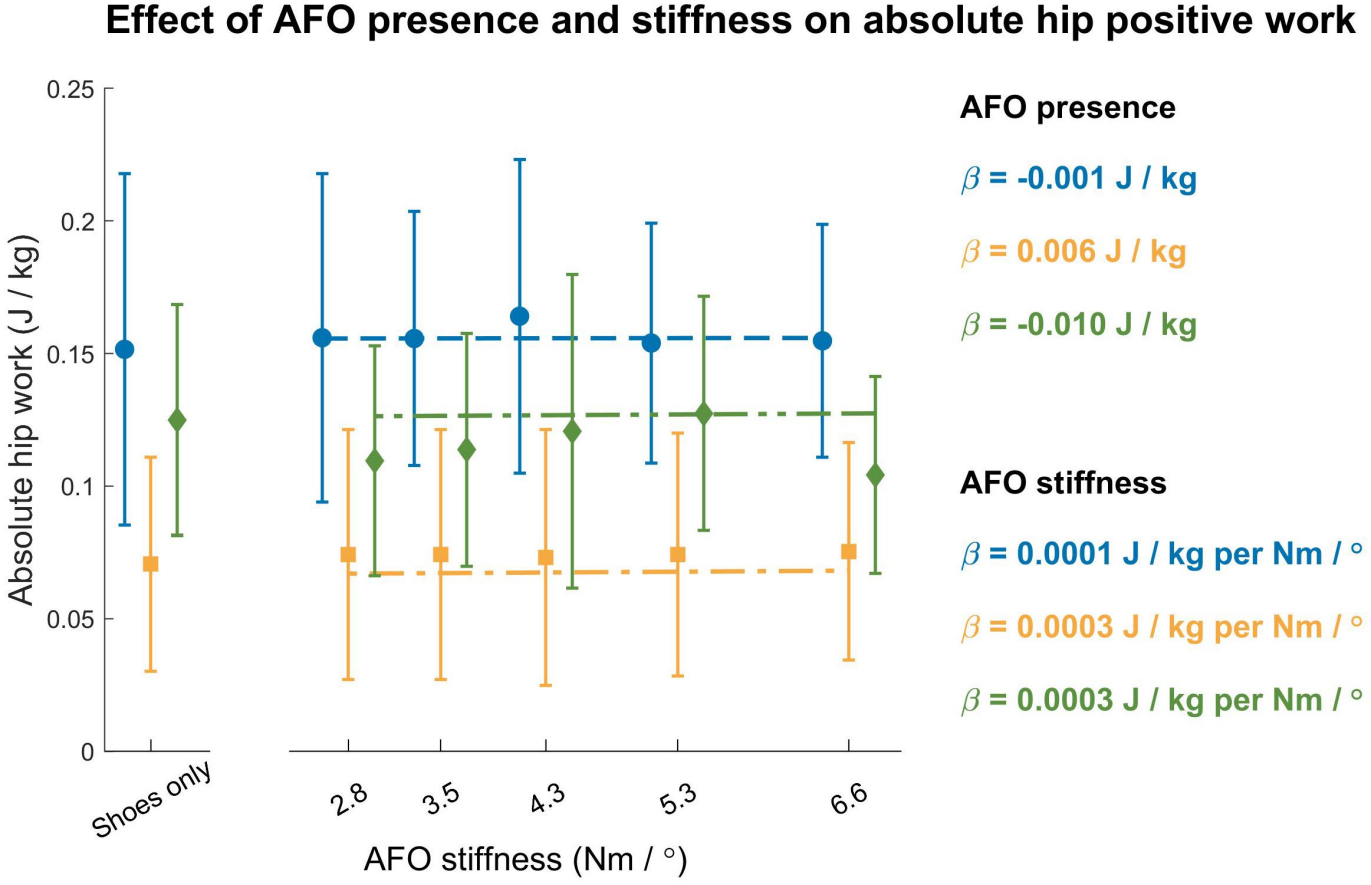
Effect of AFO and AFO stiffness on absolute hip positive work.

The AFO of 2.8 Nm/◦ did not significantly affect ankle relative positive work compared with the shoes-only condition (*F*(1*,* 127*.*4) = 0*.*90, *p* = 0*.*344, *q* = 0.459) (Figure 7). The AFO significantly reduced relative positive knee work (*F*(1*,* 126*.*7) = 13*.*41, *p* *<* 0*.*001, *q* = 0.001) (Figure 8), while relative positive hip work was increased (*F*(1*,* 130*.*3) = 9*.*97,*p* = 0*.*002, *q* = 0*.*005) (Figure 9). For all three relative joint-work outcomes, a significant main effect of movement task was found (all *q* *<* 0*.*001). Ankle work contributed relatively the most during obstacle crossing, the knee during step-up and the hip during gait initiation. No statistically significant AFO 2.8 Nm/◦ × movement task interactions were found (all *q* > 0.293). The data are shown in Table 1.

**Figure 7:**
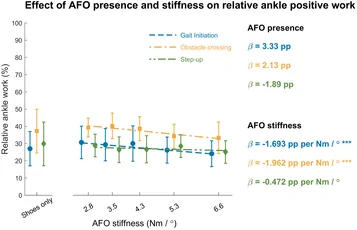
Effect of AFO and AFO stiffness on relative ankle positive work.

**Figure 8:**
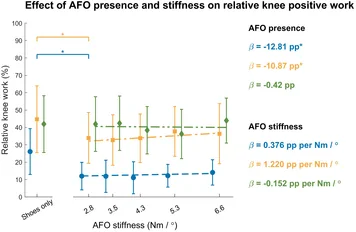
Effect of AFO and AFO stiffness on relative knee positive work.

**Figure 9:**
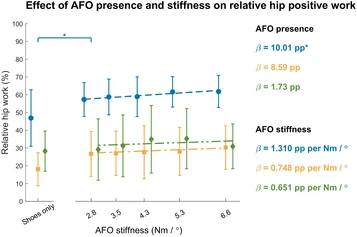
Effect of AFO and AFO stiffness on relative hip positive work.

### Effect of AFO stiffness on propulsive mechanics

AFO stiffness did not significantly affect forward impulse (*F*(1*,* 343*.*8) = 0*.*13, *p* = 0*.*721, *q* = 0.824) or upward impulse (*F*(1*,* 344*.*6) = 3*.*54, *p* = 0*.*061, *q* = 0.139). Both impulse outcomes differed significantly between movement tasks (both *q* *<* 0*.*001), but no significant stiffness  ×  movement task interactions were found (forward impulse: *F*(2*,* 344*.*3) = 1*.*66, *p* = 0*.*191, *q* = 0.510; upward impulse: *F*(2*,* 345*.*2) = 1*.*75, *p* = 0*.*175, *q* = 0.510) (Figures 2, 3).

Absolute ankle positive work decreased significantly with increasing stiffness (F(1,343.9) = 43.03, *p* < 0.001, q < 0.001) (Figure 4). No effect on knee or hip positive work was found (q > 0.398) (Figures 5, 6). A significant main effect of movement task was found for ankle, knee and hip positive work (q < 0.001 for all). Ankle and knee work were highest during step-up, while hip work was highest during gait initiation. For none of the joints a significant interaction between AFO stiffness and movement task was found (q > 0.631).

A significant main effect of stiffness was found for ankle relative positive work (*F*(1*,* 345*.*7) = 42*.*93, *p* *<* 0*.*001, *q* *<* 0*.*001), where increasing AFO stiffness reduced relative ankle work (Figure 7). A significant main effect of movement task was also found (*F*(2*,* 347*.*5) = 22*.*99, *q* *<* 0*.*001), with the highest relative contribution during obstacle crossing. The stiffness  ×  movement task interaction did not reach the FDR-corrected significance threshold (*F*(2*,* 346*.*6) = 4*.*62, *p* = 0*.*010, *q* = 0.084). No significant main effect of stiffness was found for knee relative positive work (*F*(1*,* 346*.*5) = 1*.*16, *p* = 0*.*283, *q* = 0.398) or hip relative positive work (*F*(1*,* 347*.*6) = 3*.*31, *p* = 0*.*070, *q* = 0.139) (Figures 8, 9). Both knee and hip relative positive work differed significantly between movement tasks (both *q* *<* 0*.*001). The knee contributed the most during step-up, while the hip contributed most during gait initiation. No significant stiffness  ×  movement task interactions were found for either outcome (knee: *F*(2*,* 347*.*8) = 0*.*75, *p* = 0*.*473, *q* = 0.631; hip: *F*(2*,* 348*.*8) = 0*.*18, *p* = 0*.*838, *q* = 0.958). The data are presented in Table 2.

### Functional outcomes

No significant effects of AFO stiffness were found for step length, step time, step width, movement speed, or foot clearance measures in any movement task (all *F* *<* 3*.*17, all *p* *>* 0*.*12).

## Discussion

In this explorative study no significant effects of an AFO of 2.8 Nm/◦ or increasing AFO stiffness on forward or upward impulse were found between daily-life movement tasks. Regarding joint work: an AFO of 2.8 Nm/◦ did significantly reduce ankle and knee work in absolute terms while not significantly altering hip work. Ankle relative work was not affected, while the AFO 2.8 Nm/◦ reduced relative positive knee work and increased relative positive hip work. In contrast, increasing AFO stiffness only led to a reduction in absolute and relative positive ankle work while no effects regarding knee or hip work were found. For none of the outcomes a significant interaction with movement task was found, although the stiffness-by-movement task interaction of relative positive ankle work was close to reaching significance.

Wearing an AFO of 2.8 Nm/◦ primarily altered the distribution of positive joint work across the lower limb by reducing ankle and knee work, without affecting impulses or absolute hip work. The reduction in ankle work in absolute terms but not relatively is in line with previous studies evaluating spring-like dorsal AFOs in level walking (15, 16, 19). Although spring-like AFOs support ankle power more compared to other AFO designs (20, 21), the spring-like mechanism cannot fully counteract the negative effects of the AFO on the generation of biological ankle power due to the restriction in ankle range of motion (9, 17). Our study shows that this effect is universal across multiple movement tasks. Ankle relative work did not change as knee work reduced to a greater extent. The reduction in knee work can be explained by the effect of the AFO on forward tibia progression (22). In case of calf muscle weakness the knee is forced into flexion by excessive tibial progression when the ground reaction force moves anterior of the ankle joint. AFOs restrict this tibia progression and thereby the excessive knee flexion (15). Hence, less work has to be performed to extent the knee when necessary. However, during level walking this mechanism did not result in reduced knee work (16). Potentially this is caused by the fact that knee relative work is substantially higher during the obstacle crossing and step-up compared to walking. Due to the reductions in ankle and knee work but not hip work, the relative contribution of the hip increased. True proximal compensation would require a measurable increase in absolute hip work, as demonstrated in level walking when patients walk faster with AFO and actively shift mechanical demand proximally to compensate for reduced distal output (16, 23, 24). That such increase in hip work is not found might be due to the nature of the movement task. In level walking the AFO increases walking speed substantially (16), while such improvements in task performance are likely relatively lower in gait initiation, obstacle crossing or step-up.

When evaluating the effect of AFO stiffness, we found that increasing AFO stiffness reduced ankle work, without affecting external propulsion or movement-related outcomes, such as step length. The reduction of ankle work is consistent with data of level walking where ankle power reduced to a similar extent (15). This reduction is caused by limiting ankle range-of-motion and hence rotational speed of the ankle joint (13). However, our results suggest that the effect of AFO stiffness on relative ankle work may be movement task dependent. After correction for multiple testing the interaction effect of stiffness × movement task was borderline significant. Given our small sample size this indicates a potential movement task effect. However, it should also be acknowledged that relative joint work is interdependent. A reduction in ankle work will automatically increase the relative contribution of the knee and hip joint. The magnitude of this effect depends on the relative contribution of the joint during the condition with an AFO of 2.8 Nm/◦ (most flexible AFO condition). In the future multivariate analysis may provide more insights into the relative contributions. In absolute terms the reduction was highest during obstacle crossing, but similar for gait initiation and step-up. For relative joint work gait initiation and obstacle crossing showed larger reductions, whereas limited reduction was found during step-up. This difference is explained by the mechanical role of the trailing-limb ankle in each task. During gait initiation and obstacle crossing, the trailing-limb ankle contributes relatively more compared to level walking (5). Contrary, step-up movements impose high knee and hip extensor demands for body elevation. Consequently, a reduction in absolute ankle work produced a smaller change in the ankle's relative contribution, making the stiffness effect on ankle relative work less apparent (25, 26). Nevertheless, regarding absolute ankle work there was no indication that an interaction between AFO stiffness and movement task existed. During all movement tasks ankle work reduced between 0.0051 and 0.0081 J/kg for each increase of 1 Nm/degree in AFO stiffness. This effect seems limited, however, this is approximately 5% of the absolute contribution of the AFO. Consequently, increasing the stiffness with multiple Nm/◦ will have an impact on task performance.

Across all three movement tasks, the reduction in ankle work with increasing stiffness was not accompanied by a uniform proximal redistribution of joint work. Neither relative nor absolute knee and hip positive work increased significantly across stiffness conditions. Likely this is caused by a lack of statistical power as both relative knee and hip work increase slightly. Another possibility is that compensation occurred outside the analyzed trailing-limb propulsion window or at the trunk.

Evaluating multiple movement tasks in a variety of conditions can provide highly valuable insights for clinical practice. Although we did not find significant interaction effects, relative ankle work demonstrated a tendency towards a more pronounced effect of AFO stiffness for tasks were ankle work is more dominant, such as gait initiation. Testing multiple movements that are relevant for the patient might therefore provide relevant insights. However, we did not measure energy cost or muscle stress during the movement tasks. As ankle work is essential for efficient movements and foot placement, an effect on such outcomes may be expected. Measuring them experimentally is challenging, but simulating such outcomes might provide essential insights as previously done for the sit-to-walk movement (27). Furthermore, the finding that the effect of AFO stiffness on ankle work is consistent across movement tasks also helps partly explain why incline and stair walking is more difficult with AFOs (28). Such insights are essential to better tune the stiffness to individual living circumstances in the future.

When interpreting our results, several limitations should be acknowledged. We only included 7 participants, which limits the statistical power. Due to the small sample size, results should be interpreted as preliminary findings. Despite this sample size we found several highly significant effects. However, the generalizability of the study may be limited as participants were all relatively high functioning, e.g., able to perform the tasks independently. Potentially, the effect of AFO stiffness on joint work and performance is higher in less functioning patients. Furthermore, the sample consisted of 4 males and 3 females, and their anatomical differences are known to affect how ankle push-off power is generated (29). It may therefore be possible that AFOs influence joint work differently in males and females, which should be analyzed in a larger study. Regarding the protocol it should be acknowledged that participants did not complete a separate familiarization period for each stiffness condition. Familiarization might not be required to determine biomechanical effects of AFOs and AFO stiffness, as no changes in gait biomechanics over time were found in previous studies (30–32). This may also be the case for more complex movements, especially as all participants were experienced AFO users. Furthermore, stiffness conditions were randomized, further limiting the influence of potential learning effects. Nevertheless, there might still be a bias towards the stiffness of the participants own, habitual AFO. If this is the case, participants may perform more push-off work with this stiffness level and show a difference in joint work distribution. However, as the own habitual AFO stiffness differs between participants the effect on our group analysis is expected to be small. Furthermore, other tasks of relevance such as walking on uneven ground or fast walking speed were not included, but may be of relevance. Future research should incorporate them as well. Such future studies should aim to separate biological and orthotic contributions to ankle work by combining 3D analysis, electromyography and AFO deformation measurements. This would clarify whether stiffness-related ankle work reduction reflects reduced muscle demand, altered tendon energy return, or reduced orthotic contribution. In addition, could also indiciate whether AFOs decrease metabolic cost of these movements compared to no AFO, and whether an optimal AFO stiffness exists to perform these more complex tasks. Studies with a larger sample, and with a larger AFO stiffness range should also explore non-linear relationships. Especially since the effect of 1 Nm/◦ seems larger between the shoes-only condition and 2.8 Nm/◦ compared to the effect across the different stiffness conditions (see beta's in Tables 1, 2).

In conclusion, our explorative study demonstrates that an AFO of 2.8 Nm/ and increasing AFO stiffness further reduces absolute ankle work. Despite the fact that the studied movements differed in joint distribution, no interaction with wearing an AFO of 2.8 Nm/◦or increasing stiffness was found for absolute joint work. For relative joint work our results indicate that an interaction might exist, where the effect of increasing AFO stiffness is more pronounced in activities with relatively higher ankle power demands.

## Data Availability

The raw data supporting the conclusions of this article will be made available by the authors, without undue reservation.
